# Graphene-Based Nanomaterials as the Cathode for Lithium-Sulfur Batteries

**DOI:** 10.3390/molecules26092507

**Published:** 2021-04-25

**Authors:** Jingkun Tian, Fei Xing, Qiqian Gao

**Affiliations:** School of Physics and Optoelectronic Engineering, Shandong University of Technology, Zibo 255049, China; wfcltjk@163.com

**Keywords:** graphene, lithium-sulfur battery, cathode, polysulfide, composites

## Abstract

The global energy crisis and environmental problems are becoming increasingly serious. It is now urgent to vigorously develop an efficient energy storage system. Lithium-sulfur batteries (LSBs) are considered to be one of the most promising candidates for next-generation energy storage systems due to their high energy density. Sulfur is abundant on Earth, low-cost, and environmentally friendly, which is consistent with the characteristics of new clean energy. Although LSBs possess numerous advantages, they still suffer from numerous problems such as the dissolution and diffusion of sulfur intermediate products during the discharge process, the expansion of the electrode volume, and so on, which severely limit their further development. Graphene is a two-dimensional crystal material with a single atomic layer thickness and honeycomb bonding structure formed by sp^2^ hybridization of carbon atoms. Since its discovery in 2004, graphene has attracted worldwide attention due to its excellent physical and chemical properties. Herein, this review summarizes the latest developments in graphene frameworks, heteroatom-modified graphene, and graphene composite frameworks in sulfur cathodes. Moreover, the challenges and future development of graphene-based sulfur cathodes are also discussed.

## 1. Introduction

Facing the depletion of fossil fuels and gradual serious environmental pollution problems, people have slowly realized the necessity of clean energy development. Sustainable energy such as solar and wind energy has been extensively developed [1]. However, this decentralized energy supply is not a long-term solution for social energy storage. Therefore, it is urgent to develop a stable high-capacity clean energy storage system to handle the social energy demand problem. Among the many new energy battery systems, lithium-ion batteries (LIBs) have attracted much attention due to their high discharge specific capacity, high safety, long service life, and environmental friendliness advantages [2,3].

Unfortunately, LIB cathode materials (layered metal oxides and spinel structures) are expensive and their performance has approached the theoretical limit [4,5], making it difficult to meet the long-term battery life requirements represented by electric vehicles. Hence, researching new low-cost cathode materials is an effective strategy to deal with market demand. As one of the basic elements on Earth, sulfur is abundant in nature and environmentally friendly. In particular, it has an ultra-high theoretical capacity of 1675 mAh·g^−1^ and a theoretical energy density of 2600 Wh·kg^−1^ [6,7,8,9,10]. These advantages have promoted the development of sulfur cathode materials. After decades of research, LSBs have made great progress [11,12,13]. However, there are still many problems to be solved in sulfur cathodes:

- The insulation of sulfur reduces the electron transfer rate (conductivity: 5 × 10^−30^ S·cm^−1^ at 25 °C) [14].
- The volume expansion of the sulfur cathode material after multiple electrode reactions destroys the electrode structure.
- The dissolution of soluble lithium polysulfide triggers a “shuttle effect”, which causes energy loss and low battery life.

In order to solve the above problems and achieve high electrochemical activity, a feasible and effective method is to establish abundant electron and ion transport channels inside the cathode material and provide a compatible surface for insoluble Li_2_S and S. Researchers have designed sulfur cathodes into zero-dimensional [15], one-dimensional [16], two-dimensional [17], and three-dimensional materials [18,19], rationally designing sulfur host materials from multiple dimensions to explore higher performance LSBs. Until 2009, Nazar et al. prepared a cathode composed of mesoporous carbon (CMK-3) and sulfur for the first time, obtaining an initial discharge capacity of 1000 mAh·g^−1^ at 0.1 C [20]. Their study proved the feasibility of utilizing mesoporous carbon to adsorb polysulfides. This research achievement has attracted the interest of more and more researchers and promoted the development of various forms of carbon-based materials in the field of sulfur cathodes.

Among the many carbon materials, graphene has attracted attention for its high conductivity, high specific surface area, and excellent mechanical properties. It has an electron mobility of 15,000 cm^2^·V^−1^·S^−1^ at room temperature and a theoretical surface area of 2630 m^2^·g^−1^ [21,22,23,24]. Graphene is a two-dimensional material composed of a layer of sp^2^-bonded carbon atoms [21,25]. It has excellent flexibility, which is benefit to deal with the volume change of sulfur cathode in the redox reaction and also provides a material basis for the preparation of flexible devices. Second, graphene as a matrix material can provide good conductive network support for the sulfur cathode and further improve the sulfur utilization rate. Furthermore, the programmable assembly characteristics of graphene can be flexibly changed. It not only has a large specific surface area, but also facilitates the construction of interconnected and layered macroporous networks, which is an effective method to inhibit the diffusion of polysulfides. However, non-polar graphene has a weak adsorption capacity for polar polysulfides. Graphene doping by heteroatoms can produce polar electroactive sites, which effectively overcome this shortcoming. In addition to the high conductivity and good mechanical properties of graphene, a large number of functional groups on the surface of graphene can achieve interaction with lithium polysulfides (LiPSs).

With the deep understanding of the interaction mechanism between graphene and LSBs, a variety of effective methods have been explored to prevent the shuttle effect. Correspondingly, the electrochemical performance of LSBs has also been greatly improved. In recent years, the overall structure of LSB has been summarized including cathodes, anodes, electrolytes, and separators. For instance, Pang et al. summarized the new electrolyte and intelligent cathode system in LSBs that control the dissolution of polysulfides [26]. Yang et al. and Li et al. focused on summarizing the stable performance of LSB cathode materials [27,28]. There are also examples of scientific researchers summarizing the battery from the perspective of materials. Shao et al. discussed the challenges and prospects of LSBs from the two-dimensional material level [29]. Dai et al. mainly summarized the application of graphene in the field of flexible batteries including various metal-ion batteries, metal-air batteries, and LSBs [30]. Fang et al. classified LIBs and LSBs from two materials: carbon nanotubes and graphene [31]. Wu et al. respectively summarized the development of core-shell structured S electrodes, freestanding and flexible sulfur cathodes, and functionalization of graphene-based carbon in LSBs [32]. The most recent review describing the progress of graphene in LSBs is the application of porous graphene in sulfur cathodes and lithium anodes introduced by Sun et al. [33], who looked at aspects of sulfur utilization, cathode volume change, and the reduction of lithium loss.

The above review provides a comprehensive summary of the overall structure of LSBs. However, it should be noted that a comprehensive review of graphene as cathodes for LSBs is still in progress. In this review, we first systematically describe the interactions between sulfur and graphene, and summarize the structure of the sulfur cathode using the pure graphene framework studied in recent years. Furthermore, taking the graphene structure as the main line, the electrochemical performance improvement of sulfur cathodes has been improved in more detail from the fields of heteroatom-modified graphene, metal compound-modified graphene as well as other carbon materials and graphene composite materials.

## 2. Graphene as the Positive Electrode Skeleton

Since graphene was mechanically exfoliated by Geim et al. in 2004 [34], the preparation methods, characterization methods, and physical and chemical properties of graphene have been extensively developed. These important studies laid the foundation for the application of graphene in electrode materials. Since then, the superior conductivity and higher chemical stability of graphene have been widely recognized by scientific researchers. Graphene-based materials as the positive electrode framework of LSBs have made rapid progress in recent years. Its relationship with sulfur and its advantages and disadvantages as a pure cathode framework are introduced and summarized in this section.

### 2.1. The Interactions between Sulfur and Graphene

**Figure 1:**
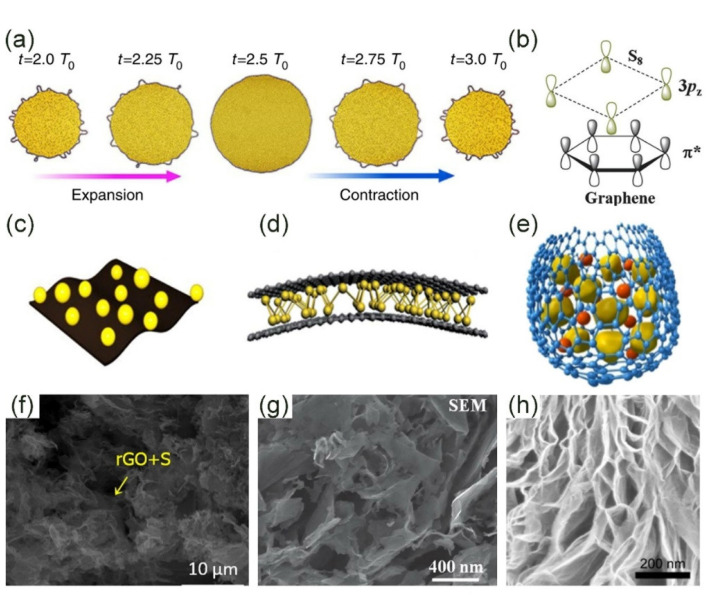
Interaction between graphene and sulfur and its configuration. (**a**) Macroscopic volume change of graphene-coated sulfur particles [35]. Copyright 2014, Springer Nature. (**b**) Microscopic symmetry and non-polarity of graphene and sulfur [36]. Copyright 2013, Royal Society of Chemistry. (**c**) The in-plane structure has the advantage of flexible electrodes [38]. Copyright 2016, Elsevier. (**d**) Sandwich structure: sulfur is confined between two or more layers of graphene sheets [36]. Copyright 2013, Royal Society of Chemistry. (**e**) Core-shell structure: sulfur and LiPSs are coated by graphene to prevent leakage [39]. Copyright 2013, Elsevier. (**f**) Scanning Electron Microscope (SEM) image of complex of sulfur and graphene oxide (S/GO) [38]. Copyright 2016, Elsevier. (**g**) The sandwich structure of sulfur is evenly distributed on the graphene sheet [36]. Copyright 2013, Royal Society of Chemistry. (**h**) SEM image of core-shell structure [39]. Copyright 2013, Elsevier.

There are both physical and chemical interactions between sulfur molecules and graphene and these two interactions complement each other. In terms of physical interactions, first, graphene’s various geometric features act as a substrate material for coating sulfur particles on a macroscopic scale (Figure 1a) [35]. Besides, its flexibility is also suitable for electrode applications. The stretching ability of the sulfur cathode framework is essential for the improvement of electrochemical performance. In fact, graphene boosts the charge transfer between sulfur particles and electrolytes due to its unique structure. The physical coating of graphene can prevent the spreading of dissolved polysulfides. Porous graphene can accommodate the volume expansion and improve the utilization of sulfur during the charge–discharge process. Moreover, graphene is a hexagonal honeycomb lattice structure composed of carbon atoms with sp² hybrid orbitals. The S_8_ molecule is a zigzag ring with eight sulfur atoms, which is called a double-layer octagonal structure. The crystal structures of graphene and element S_8_ molecules are both highly symmetrical and both possess non-polar properties, as shown in Figure 1b. Therefore, van der Waals forces are very strong. The interaction between the two is reflected in the lone pairs of the S 3p_z_^2^ electrons and the antibonding conjugated π* states of the graphene plane [36,37]. Intriguingly, during the discharge process, the larger the electron density of the polysulfide, the stronger the above interaction will be. Therefore, a graphene-based material can not only immobilize the element S_8_ molecule, but also fix LiPSs, which is a good choice for the sulfur cathode framework.

### 2.2. Configurations of Pure Graphene and Sulfur

In recent years, a variety of graphene/sulfur configuration materials have been developed as sulfur cathodes. In order to maximize the utilization of active materials, researchers have studied the graphene/sulfur configuration from a variety of perspectives. The basic configuration is divided into the following types: in-plane, three-dimensional sandwich, three-dimensional core-shell, and so on. This section summarizes the various configurations of sulfur cathodes based on unmodified graphene.

There are several forms for in-plane: graphene sheets, graphene paper, graphene nanoribbons, etc. [40,41,42,43,44]. Their names are different according to different preparation methods. Among them, graphene nanoribbons are the most special. Graphene nanoribbons are intermediary products of graphene and carbon nanotubes. The graphene nanotubes can be cut and expanded longitudinally to obtain graphene nanoribbons. Compared with other paper-like structures, graphene nanoribbons are composed of a large number of quasi-one-dimensional graphene nanoribbons that are closely connected to each other, which is beneficial to improve the stability of the electrode. The self-assembly process induced by water evaporation was developed by Liu et al. [40]. The obtained graphene nanoribbons made the internal structure network tightly connected due to the benefits of evaporation, which can not only promote charge transfer but also physically limit LiPSs. In-plane means that the redox reaction takes place on the surface of the graphene paper (Figure 1c). Therefore, with the progress of the reaction, the inner part of the graphene paper surface is etched, and the intermediate pore and fold structure are gradually formed, thus increasing the specific surface area of the material (Figure 1f) [42,43,44]. The advantage of the in-plane structure is that its flexible electrode can also show good electrochemical performance in the bent state. Moreover, the utilization rate of the graphene/sulfur active material with a paper-like structure is very high. However, it is a flat structure after all and cannot achieve the effect of macro-physical packaging.

**Figure 2:**
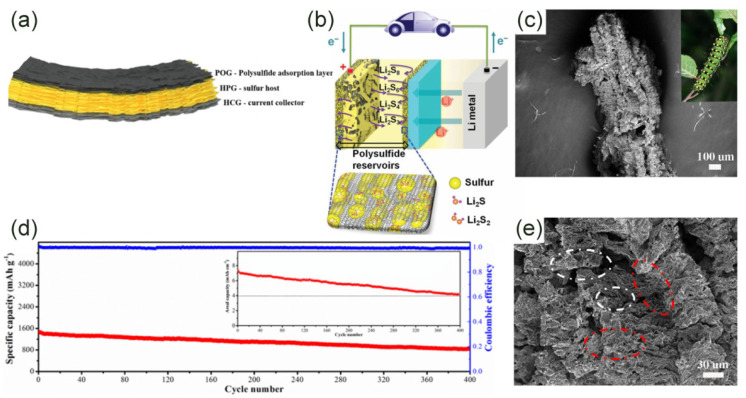
(**a**) Schematic diagram of all-graphene sandwich structure [48]. Copyright 2016, American Chemical Society. (**b**) Electrode configuration diagram: LiPSs are confined between graphene and diaphragm [45]. Copyright 2014, Springer Nature. (**c**) SEM image of caterpillar-like graphene [49]. Copyright 2017, Elsevier. (**d**) The long cycle test and the corresponding area capacity of the sulfur cathode at 0.34 A·g^−1^ [48]. Copyright 2016, American Chemical Society. (**e**) Layered (red circles) porous (white circles) structure shown by SEM image [49]. Copyright 2017, Elsevier.

The sandwich structure is a simple physical packaging scheme, and the sulfur is confined between two or more layers of graphene nanosheets (Figure 1d,g) [45,46,47,48]. Recently, Li et al. wrapped ultrafine nano-sulfur particles between two layers of graphene to form a sandwich structure [46]. Ultra-fine sulfur particles have a larger electronic contact area than bulk materials, avoiding the “dead sulfur” problem. The electrode material’s capacity of 1208 mAh·g^−1^ at 0.1 C was also due to the effective physical limitation of the sandwich structure. Fang et al. prepared a full graphene sandwich structure composed of high-porosity graphene (HPG), highly conductive graphene (HCG), and partial graphene oxide (POG) (Figure 2a) [48]. Sulfur was mainly in HPG. HCG was used as a current collector and POG was used as an adsorption layer for polysulfides. Three kinds of graphene played their respective roles, making the initial discharge capacity as high as 1500 mAh·g^−1^ at 0.34 A·g^−1^. Surprisingly, the electrode still had an area capacity of 4.2 mAh·cm^−2^ after 400 cycles (Figure 2d). There was also a very special sandwich structure in which sulfur and LiPSs were confined between the graphene and the separator. Graphene was coated on the sulfur and separator, respectively (Figure 2b). This structure adapts to the volume expansion in the lithiation process to a greater extent. Graphene on the surface of the separator also effectively reduces the shuttle effect [45]. Obviously, the sandwich structure has excellent electron transfer capabilities and can also alleviate volume shrinkage/expansion to a certain extent. However, for a long-term cycle, the dissolved polysulfides will inevitably leak from the edge of the graphene, which is very detrimental to the battery life.

The core-shell structure has been proven to be able to solve the above-mentioned problems of LiPS leakage and poor volume buffer capacity. Graphene is used as an outer packaging to firmly bind the sulfur particles in the shell (Figure 1e,h). The LiPS intermediate product of the redox reaction cannot break the boundaries of graphene packaging. In addition, the core-shell structure is often very flexible, and has a good ability to deal with the volume shrinkage/expansion caused by the electrode reaction [39,50]. Although the core-shell structure can solve the problem of volume change, the large accumulation of sulfur particles in the center of the shell inevitably reduces the electron transfer rate between lithium and sulfur, and the synthesis process of the core-shell structure is complicated and cumbersome.

In order to obtain better electrochemical performance, researchers are more willing to effectively combine three-dimensional, in-plane, sandwich, core-shell, and other structures, even though the synthesis process is complex and cumbersome. For instance, Yoo et al. transformed graphene nanosheets with an in-plane structure into a columnar structure of graphene nanorolls through a freeze-casting process [38]. This structure combines the characteristics of in-plane and sandwich. Sulfur is wrapped inside the nano-volume to act as a physical barrier to the diffusion of LiPSs. A similar structure is caterpillar-like graphene (Figure 2c), which is different from graphene nano volumes in that it has a layered, dense porous, and wrinkled structure inside (Figure 2e) [49]. In particular, the dense porous structure enables the track-like graphene to contain more sulfur particles. Similar to the core-shell structure is a microsphere-type graphene structure, where the combination of sandwich structure and core-shell structure is characteristic. The rod-shaped nano-sulfur is uniformly deposited in the reduced graphene oxide structure by spray freezing and the combination of spray-freezing components and ozonation is used to control the size and pore structure of the microspheres [51]. Therefore, this structure achieves a higher sulfur utilization rate. Graphene aerogels also have good electrochemical benefits as an electrode material. A reduced graphene oxide aerogel as a stable interconnected porous conductive scaffold can promote the reaction of Li^+^ with polysulfides. The oxygen-containing groups and a large amount of space in the rGO aerogel inhibit the migration of LiPSs from the cathode [52,53]. The graphene framework of these multiple configurations is stronger than the graphene framework of a single configuration in terms of rate capability and cycle capability. However, its complicated preparation procedure also inevitably increases the cost.

## 3. Heteroatom Doped Graphene

The hydrophobic structure of the graphene sulfur cathode is exactly contrary to the hydrophilicity of LiPSs, and the well-known van der Waals force interaction cannot retain LiPSs. Consequently, it is far from enough to rely on a simple graphene structure to achieve good electrochemical performance. Heteroatom-modified graphene is an interesting method that can change the polarity of graphene materials and provide active sites for polysulfides. Therefore, the affinity between the graphene-based sulfur cathode and LiPSs is greatly improved. In this section, we have summarized the electrochemical performance of monoatomic and diatomic modified graphene-based sulfur cathodes in LSBs, and present them in Table 1.

### 3.1. Single-Atom Doped Graphene

Single-atom doped graphene has been developed for sulfur cathodes for many years such as nitrogen (N), boron (B), oxygen (O), phosphorus (P), and sulfur (S). They form a chemical bond with LiPS to prevent it from diffusing to the lithium anode to inhibit the shuttle effect. Since they have different chemical bond energies with LiPSs, their adsorption capacity is also different. Therefore, this section only summarizes the more popular nitrogen atom doping and boron atom doping research progress in recent years.

#### 3.1.1. Nitrogen Doping

Nitrogen-doped graphene (NG) has been researched for several years. In early work, Reddy’s research group introduced N atoms into the graphene structure as a graphene electrode by inducing surface defects [67]. The test results showed that the reversible discharge capacity of NG electrodes was twice that of pure graphene electrodes. This conclusion has attracted the attention of a large number of researchers. This research provided a new strategy for the development of high-performance graphene electrodes. Doping nitrogen atoms into carbonaceous materials can improve lithium storage capacities. Therefore, in the past few decades, nitrogen-doped carbon material has caused serious concerns for researchers [68,69]. At present, in the field of energy storage, especially in terms of electrode materials for LSBs, the most studied single-atom doped graphene is NG. Through nitrogen doping, the transport speed for electrons and lithium ions in graphene is promoted. At the same time, the sulfur and nitrogen atoms in the graphene electrode are more strongly fixed due to strong chemical bonding, which can inhibit the loss of sulfur on the surface of graphene. For example, Tang et al. emphasized that nitrogen has the advantages of fixing sulfur and controlling volume expansion [70]. NG material is added with sulfur by the melt diffusion method, the content of pyridinic-N is reduced, and part of the carbon–nitrogen double bonds become single bonds accompanied by the bonding of N–S, so that the sulfur is stably present in the graphene shell. When the temperature reaches 155 °C, the sulfur that does not interact with the N element on the graphene shell through bonding will sublime to control the sulfur content and deal with the volume expansion of the sulfur cathode.

**Figure 3:**
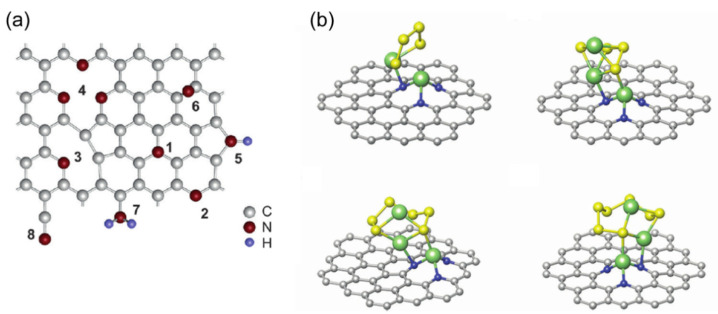
(**a**) Configuration of nitrogen-doped graphene: (1) graphitic-N, (2) pyridinic-N, (3) single N pyridinic vacancy, (4) triple N pyridinic vacancy, (5) pyrrolic-N, (6) interstitial N or adatom, (7) amine, (8) nitrile [72]. Copyright 2011, American Chemical Society. (**b**) The adsorption configuration diagram of LiPSs in LiNG. The gray, blue, yellow, and green spheres represent the C, N, S, and Li atoms, respectively [76]. Copyright 2017, Royal Society of Chemistry.

**Figure 4:**
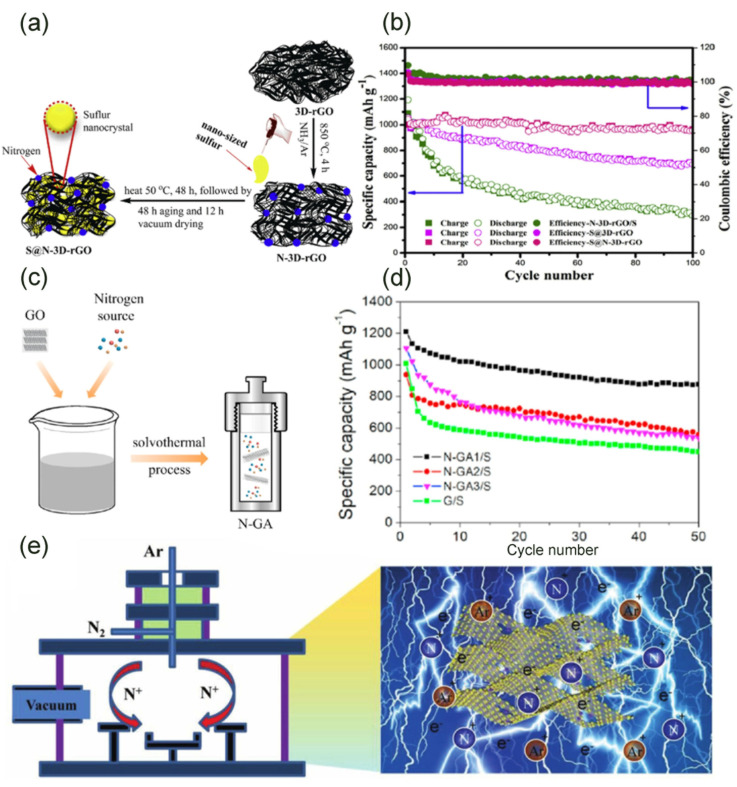
Several methods of NG and the performance of cathode. (**a**) Synthesis of S@N-3D-rGO. (**b**) Cyclic stability and coulombic efficiency of sulfur cathode at 0.2 C [54]. Copyright 2017, Elsevier. (**c**) Hydrothermal synthesis diagram of NG. (**d**) Cyclic performance of sulfur cathode at 0.1 C [57]. Copyright 2021, Elsevier. (**e**) Synthesis diagram of NG by hollow cathode discharge method [55]. Copyright 2019, John Wiley and Sons.

**Figure 5:**
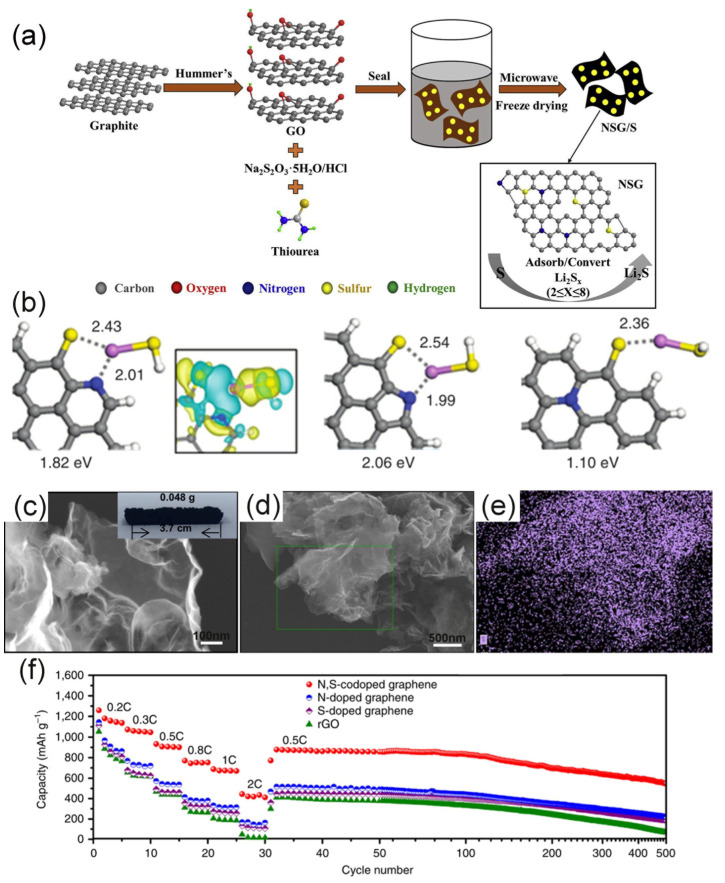
(**a**) Schematic diagram of one-pot synthesis of NSG/S [60]. Copyright 2018, Elsevier. (**b**) The binding energy of Li in LiPSs and N/S co-doped graphene between pyridine-N, pyrrole-N, and unbound N. Grey, white, blue, yellow, and purple balls represent C, H, N, S, and Li atoms, respectively [61]. Copyright 2015, Springer Nature. Morphology and microstructure of N, S-FLG900 (**c**) and N, S-FLG900/S (**d**) FESEM images and sulfur mapping (**e**) [86]. Copyright 2018, Royal Society of Chemistry. (**f**) Cycle performance test of various sulfur cathodes [61]. Copyright 2015, Springer Nature.

In the basic research of the N and LiPS adsorption system, N-doping can effectively adsorb LiPSs due to the strong attraction between N and the less electronegative Li^+^. The adsorption energy of S_8_ on simple graphene and NG has no obvious change, indicating that there is no N–S interaction in the adsorption system. N-doping shortens the bond distance between Li^+^ and carbon, and LiPSs are captured as a whole molecule [54,71]. N-doping has been summarized in many types. As shown in Figure 3a, eight doping forms were summarized by Usachov [72]. Currently, the doping forms of N used as adsorption sites are mainly pyridinic-N (Figure 3a-2), pyrrolic-N (Figure 3a-5), and graphitic-N (Figure 3a-1). Due to the different positions of nitrogen atoms, their electron density structures are also different, so they play different roles. The pyridinic-N (398.3 eV) is bound to the two carbon atoms at the edge of the sp^2^ structure and has a lone pair of electrons in the plane of the ring. Therefore, this active site improves the binding energy of the graphene framework and LiPSs. Because the band gap of the highest occupied molecular orbital (HOMO) and the lowest unoccupied molecular orbital (LUMO) is reduced, the greater effect of graphite N (401.4 eV) is to increase the conductivity of the graphene framework [54,73]. Many studies have shown that pyridinic-N and LiPSs have significant interaction. In the NG materials prepared by Li et al. [74], it is believed that the clustered pyridinic-N dopant is an important factor in binding LiPSs. Sun et al. prepared nitrogen-enriched mesoporous carbons with tunable nitrogen content material for LSBs [75]. In the discharge process, overcharge capacity decreases with increasing nitrogen content. A typical feature of the polysulfide shuttling phenomenon is the increase in overcharge capacity. Therefore, they proved that basic nitrogen atoms can be used as an immobilizer for anchor polysulfide anions. Meanwhile, it also proved that the nitrogen atom in pyridine and polysulfide have a strong interaction.

The shuttle effect is believed to be caused by the higher solubility of higher-order LiPSs in the electrolyte than lower-order LiPSs [9,76]. Yi proposed a new type of Li-trapped N-doped graphene (LiNG) structure and further studied the binding energy of high-order LiPSs on LiNG substrates (Figure 3b) [76]. The LiNG substrate originally contained trapped Li^+^, which is equivalent to the formation of new positive active sites. Although the Li^+^ in the substrate and the Li^+^ that is about to be adsorbed (existing in LiPSs) are positively charged and repel each other, the S atoms in LiPSs are negatively charged, thus forming a new S_molecule_–Li_substrate_ interaction. The binding energy resulting from this new interaction is always greater than the binding energy of LiPSs and electrolytes (DOL (1,3-dioxolane) and DME (1,2-dimetylethane)). Therefore, LiNG effectively inhibits the dissolution of LiPSs. The proposed LiNG structure is a further extension of NG in LSBs, indicating that there are still many new knowledge points waiting to be explored in the NG system.

NG is generally obtained by the thermal annealing of graphene with mixed nitrogen source gas [56,74,77,78]. Zegeye et al. used this method to obtain a nitrogen-doped three-dimensional reduced graphene oxide sulfur cathode (S@N-3D-rGO) (Figure 4a) [54]. Due to nitrogen doping, the pore volume of cathode material increased seven times and the sulfur content reached 80%. Sulfur particles were uniformly distributed in S@N-3D-rGO without obvious aggregation and the initial discharge capacity at 0.2 C was 1042 mAh·g^−1^, while the capacity retention rate reached 94.8% after 100 cycles (Figure 4b). Similarly, Song et al. obtained NG with a large pore volume (5.4 cm^3^·g^−1^) through an improved heat treatment method (cyanimide was used as N source and porogen) [77], and the sulfur content reached 90 wt%. This provides an effective preparation method for NG with high sulfur loading and large pore volume. The surface of NG was highly crumpled, which effectively prevented the restacking of graphene sheets. Li et al. heat-treated ammonia and argon separately, and the resulting NG sulfur electrode was subjected to 300 cycles at 0.75 A·g^−1^ [74], and the capacity decay of each cycle was only 0.05%. Duan et al. treated GO with a hollow cathode discharge (HCD) plasma in a stream of argon and nitrogen (Figure 4e) [55]. This simple and effective strategy made the graphene skeleton a porous structure, increased the sulfur content to 90 wt%, and provided a specific discharge capacity of 1186 mAh·g^−1^ at 0.1 C.

The high temperature of the heat treatment method may destroy the C–N bond. The solvothermal method is also an effective method that is commonly used to prepare NG. This method has the characteristics of low temperature and simple operation. For example, Cui et al. obtained NG using a mixture of GO and urea by a simple hydrothermal synthesis method [56]. Similarly, Jia et al. also used the hydrothermal method to prepare NG aerogels using ethylenediamine, urea, and ammonia as N sources to obtain N-GA1, N-GA2, and N-GA3 (Figure 4c) [57]. Although the pyrrolic-N ratio was different, the pyridinic-N of N-GA1 was almost twice that of N-GA2, and the initial discharge capacity of N-GA1/S at 0.1 C was 1210.7 mAh·g^−1^ (Figure 4d); cycle performance was also better than the other two. Their research results proved the advantages of pyridinic-N in reducing the loss of active materials and improving battery stability. It is worth mentioning that Yanilmaz et al. invented a graphene doping method that could control the percentage of pyridinic-N and pyrrolic-N. The way to control the content is to adjust the power and time of plasma treatment [79]. This purpose-oriented doping method is rare in scientific research. However, due to the strict requirements of experimental equipment and the environment, this method has not been well developed in the field of LSBs.

At present, there are two main problems in the field of NG. On one hand, the concentration of N in NG is low, resulting in insufficient active sites and adsorption of LiPSs. On the other hand, the N atoms in NG usually coexist in multiple forms, and the specific reasons for the strength of the adsorption performance caused by the doping site are not clear in the microscopic field. Therefore, there are still many challenging problems to be studied in the application of NG in LiSBs.

#### 3.1.2. Boron Doping

In addition to nitrogen doping, boron-doped graphene can also be used to prepare LSB cathodes with better cycling performance. B-doping is considered to be an effective way to increase conductivity and improve battery rate performance [80]. The boron atom has one extranuclear electron less than the nitrogen atom [81], which means that the boron atom can easily combine with anions on the carbon skeleton to form a strong chemical bond. Chemical adsorption can be used to fix polysulfides. It has been proven that chemical adsorption is stronger than porous physical adsorption and has a stronger effect on inhibiting the diffusion of polysulfides and improving the utilization of active materials [58,82].

Yang reported a B-doped graphene aerogel [58]. Their research compared B-doped graphene aerogel (BGA) cathodes, N-doped graphene aerogel (NGA) cathodes, and pure graphene aerogel (GA) cathodes. The results showed that the BGA-S cathode had better electrochemical performance. Sulfur has the highest binding energy in BGA-S, because doped boron has a stronger adsorption effect on polysulfides, and the gas generated by the discharge was captured by BGA. The process did not occur in GA-S and NGA-S. In the electrochemical performance test of BGA-S materials, when the current density was 0.2 C, the initial discharge capacity of BGA-S was 1290 mAh·g^−1^. After 100 cycles of the discharge test, it still maintained 994 mAh·g^−1^. However, under the same conditions, the NGA-S cathode only maintained 572 mAh·g^−1^ after 100 cycles of discharge tests, and the capacity attenuation was severe. The research also compared the current densities of 0.1 C, 0.2 C, 0.5 C, 1 C, and 2 C. The results showed that the electrochemical performance of the BGA-S cathode was better. Such high performance was attributed to the strong adsorption of B atoms on polysulfides, which reduced the shuttle effect while increasing the capacity. Zhou et al. also studied the effect of N-doped and B-doped on the performance of graphene aerogel electrodes [82]. Unlike the above, they coated Li_2_S on graphene aerogels doped with different atoms through a liquid infiltration-evaporation coating method, respectively. The cycle performance was tested at a current density of 0.3 C. The initial discharge capacity of Li_2_S/N-doped graphene electrode and Li_2_S/B-doped graphene electrode was 801 mAh·g^−1^ and 720 mAh·g^−1^, respectively. When the current density was 0.5 C, after 300 charge/discharge cycles, the capacity remained 430 mAh·g^−1^ and 357 mAh·g^−1^, corresponding to the capacity decay rate per cycle 0.129% and 0.134%, respectively. They pointed out that the adsorption process of B-doped graphene is mainly due to the adsorption of LiPSs by the B–S bond. Compared with NG, a small amount of B content helped to improve electrochemical performance, which is contrary to N.

Recently, the preparation of heteroatom-doped graphene by the electrochemical exfoliation method has received widespread attention. Yang obtained N-doped graphene by the electrochemical exfoliation method in natural biocompatible glycine (H_2_NCH_2_COOH) and ammonia aqueous solution [83]. Benjamin also prepared nitrogen-doped graphene by the same method [84]. Inspired by this, Yang et al. prepared B-doped graphene sheets (B-EEG) with a boron content of 1.866% using the electrochemical exfoliation method [59]. The S@B-EEG electrode with a sulfur level of 72.5% had an initial discharge capacity of 1476 mAh·g^−1^ at 0.1 C, and a discharge capacity of 801 mAh·g^−1^ at a rate of 0.5 C. In the LiPSs adsorption test, the electrolyte solution with B-EEG had a stronger ability to capture polysulfides.

It can be seen that B-doped can indeed increase a certain capacity and cycle stability due to the strong interaction with LiPSs from the data obtained by the above researchers. In recent years, there have been a few reports on the application of pure B doped graphene electrode in LiSBs due to its complicated preparation process, high cost, and difficulty in the accurate characterization of B atoms. The specific chemical mechanism caused by boron doping needs further theoretical explanation.

### 3.2. Dual Doped Graphene

Single-atom modified graphene has been proven to fix polysulfide well, but its binding effect is limited. In order to better suppress the shuttle effect, researchers have proposed doping dual atoms into graphene. Nitrogen-sulfur co-doping, nitrogen-boron co-doping, nitrogen-oxygen co-doping, single-atom metal, and nitrogen co-doping are several forms of doped graphene that have become popular in recent years.

#### 3.2.1. N/S Dual Doped

The adsorption force of monoatomic S-doped graphene on LiPSs is not obvious. When graphene is co-doped with S and N, its inhibitory effect on polysulfides is greatly improved. This synergy is mainly attributed to the spin effect of N atoms, which promotes the combination of S atoms and LiPSs, and the reversible conversion between polysulfides. N- and S-atoms doped on a graphene lattice can introduce active sites to increase the active regions on the surface of the graphene material. The LiPSs generated during the charging/discharging process are inhibited by the active regions on the surface of the material. The sulfur atoms and polysulfides form new sulfate groups that can act as new polysulfides and provide additional capacity [60,61,71,85].

The interaction between N/S co-doped graphene and LiPSs is stronger than that of single atom doped graphene. Zhou proved this view through density functional theory (DFT) [61]. They compared the bond energy of N/S single-atom doped and N/S dual doped graphene. The binding energies between Li in LiPSs and N/S co-doped graphene with pyridinic-N, pyrrolic-N, and uncombined-N were 1.82, 2.06, and 1.10 eV (Figure 5b) respectively. These binding energies are higher than those of N and S single atom doped graphene. The C–S bond has been mentioned in many articles [79,81,82]. The mismatch of the outermost orbitals of S and C causes the spin density of the doped graphene to be relatively dispersed. The electronegativity of S is 2.58, and the electronegativity of C is 2.55, so S has a certain electrocatalytic effect on the graphene substrate.

Based on the above chemical theoretical study of N/S co-doped graphene, researchers have co-doped N and S atoms into carbon-based materials such as graphene sponge, graphene nanosheets, and multilayer graphene [60,61,62,63,86]. The graphene sponge designed by Zhou has a 3D interconnected network structure with rich pores up to 2–8 μm in size [61]. This structure is not only beneficial to the rapid diffusion of ions, but can also be used as the adsorption storage layer of polysulfides. This structure can achieve a high specific capacity of 1200 mAh·g^−1^ at 0.2 C, with excellent cycle stability and a coulombic efficiency close to 100%. It is noteworthy that they proved that N/S co-doped graphene sulfur cathode possessed better electrochemical performance than N or S single-atom doped materials (Figure 5f). Graphene nanosheets with large specific surface area have attracted the extensive attention of researchers. However, due to the van der Waals interaction, the nanosheets will aggregate during the preparation of the electrode, resulting in the absence of active sites on the surface of the nanosheet. Therefore, the large specific surface area cannot be fully utilized [87]. Xu et al. solved the above-mentioned problems through co-doping of N and S and porous structure and improved the adsorption rate of LiPSs [63]. When it is used as the cathode host, the specific capacity is 1178 mAh·g^−1^ at a current rate of 0.2 C, and the retention capacity after 600 cycles is 780 mAh·g^−1^.

Yuan synthesized a N–S co-doped graphene platelet electrode (NSG/S) with a mesoporous architecture, which is beneficial to the uniform dispersion of sulfur in the mesopores [62]. The initial discharge capacity of NSG/S reached 1635 mAh·g^−1^ at 1 C, and also maintained good cycle reversibility at high current rates (retained at 716 mAh·g^−1^ after 100 cycles at 2 C). This reflects the importance of N and S functional groups and microstructure on the performance of the battery. Electrode preparation methods also play an important role in battery stability. For example, the NSG/S prepared by Chen using the one-pot microwave-assisted method was 819 mAh·g^−1^ after 100 cycles at 0.1 C, showing a better specific discharge capacity [60]. The preparation method of NSG/S is shown in Figure 5a. Graphene is a great adsorption material, combined with the characteristics of high microwave heating speed, uniform heating, and high efficiency. Therefore, the heteroatoms can be more firmly integrated into the graphene lattice. Li indicated that the calcination temperature had a crucial influence on the specific surface area and pore volume of graphene [86]. This phenomenon was attributed to the effect of temperature on the electrode material. The temperature sensitivity of the material was used to control the surface profile of graphene and the doping level of heteroatoms (Figure 5c is a graphene wrinkle structure and Figure 5d,e shows the uniform distribution of sulfur). The prepared synchronous-doped few-layer graphene cathode (N, S-FLG/S) can provide a reversible capacity of about 506.4 mAh·g^−1^ at a current density of 1.6 A·g^−1^ after 500 cycles.

Up to now, there has been little research on N/S co-doping graphene in electrodes. Recent studies have shown that N/S co-doping can show good performance in LSBs. N/S co-doping not only improves the conductivity of the material, but also increases the affinity of the graphene framework with LiPSs. Referring to the valuable experience of the above researchers, the stability of electrode materials can be greatly improved by preparing electrode materials by adjusting the parameters.

#### 3.2.2. N/B Dual Doped

**Figure 6:**
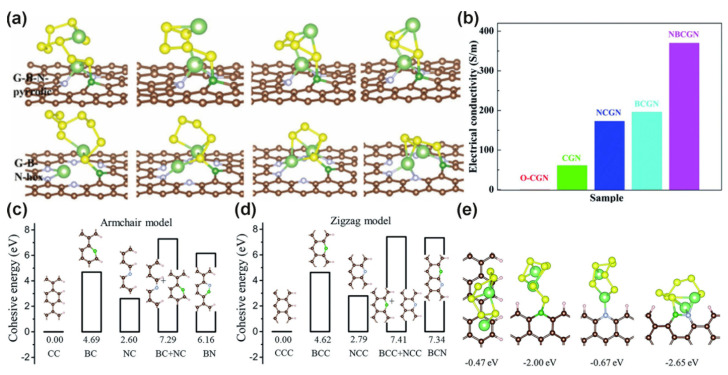
(**a**) Adsorption configurations of Li_2_S_n_ (n = 3, 4, 5, 8) clusters on the G–B–N–pyrrolic and G–B–N–hex systems [88]. Copyright 2016, Royal Society of Chemistry. (**b**) Comparison of conductivity of graphene composites. Cohesive energy for each system of the armchair model (**c**) and zigzag model (**d**). Optimized adsorption structure of Li_2_S_6_ on the pristine graphene edge, B doped system, N doped system, and N and B co-doped system as well as their binding energies (**e**) [64]. Copyright 2017, Royal Society of Chemistry.

Numerous studies have shown that the doping type of different atoms has varying degrees of influence on the binding energy of LiPSs. N/B co-doped graphene mainly inhibits the diffusion of LiPSs through the chemical combination of B–S and N–Li interaction. However, the effect of doping N and B contents and the percentage of N–B structure on polysulfides is still lacking. Li et al. systematically investigated the influence of N/B co-doping on the immobilization of LiPSs [88]. Their study found that different configurations of G–B–N (G–B–N–tri, G–B–N–rect, G–B–N-pyrrolic, and G–B–N-hex represent the G–B–N of triangle, rectangle, pyrrole, and hexagonal defects, respectively) adsorbed the Li_2_S_6_ system with different local density of states (LDOS). Excessively doped B atoms would destroy the Li–N interaction to a certain extent, but strengthen the B–S interaction. The adsorption model in Figure 6a shows that G–B–N–pyrrolic and G–B–N-hex had the strongest S–B effect, and their contribution to alleviating the “shuttle effect” was relatively large. They also found that the adsorption level of polysulfides varied with the chemical environment of B and N co-doped vacancies. As for the B–N–G systems, the interaction of pyrrolic-N with Li–N is stronger than that of pyridinic-N. This is completely opposite to the bonding effect in N-doped graphene.

**Figure 7:**
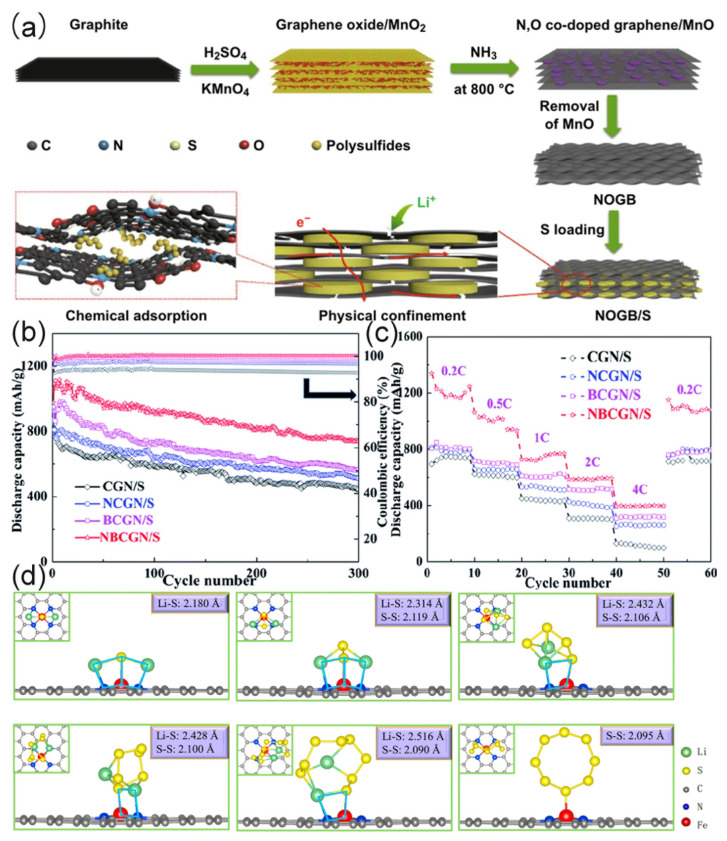
(**a**) Preparation process of the NOGB/S composite material [65]. Copyright 2020, Springer Nature. (**b**) Cycle performance and coulombic efficiency of electrode materials at 0.2 C. (**c**) Rate capabilities of different sulfur cathodes [64]. Copyright 2017, Royal Society of Chemistry. (**d**) Top and side view of the optimized lowest-energy adsorption configurations of Li_2_S_n_ (n = 1, 2, 4, 6, 8) and S_8_ on the FeN_4_@graphene surface [89]. Copyright 2019, Elsevier.

On the basis of simply studying the bond sites of N/B co-doped graphene, Chen et al. applied the N/B co-doped graphene to LSBs [64]. They used urea and boric acid as heteroatom sources to prepare curved graphene nanoribbons (NBCGN) co-doped with N and B with a larger specific surface area through a hydrothermal process. In addition, the conductivity of curved graphene nanoribbons (CGN), oxidized curved graphene nanoribbons (O-CGN), N-doped curved graphene nanoribbons (NOCGN), B-doped curved graphene nanoribbons (BOCGN), and NBCGN were compared by a four-probe technique. The results are shown in Figure 6b. Single-atom doped CGN can improve the conductivity, but the conductivity of NBCGN has the most obvious advantage, reaching 370 S·m^−1^. From the dopant amounts of nitrogen and boron, the armchair model (N–B directly bonding) in Figure 6c and the zigzag model (N–B indirectly bonding) in Figure 6d indicate that graphene doped with N and B showed a high doping amount. Their research also studied the difference between N–B adsorption of polysulfides on specific chemical bonds. The equation E_b_ = E_sys_ − E_sub_ − E_mol_ indicates that the adsorption force with the highest negative binding energy is the strongest. Taking Li_2_S_6_ as an example, Figure 6e clearly shows that N and B co-doped graphene had the highest negative binding energy and the strongest adsorption effect on Li_2_S_6_. It has been proved that the neighboring N–B structure can be tightly combined with polysulfides. Therefore, polysulfides will not diffuse too much into the electrolyte. The performance of the NBCGN/S cathode was better than that of the single-atom doped cathode whether in the long-cycle test (as shown in Figure 7b) or cathode rate performance (as shown in Figure 7c). The NBCGN/S cathode demonstrated a steady discharge capacity of 1200 mAh·g^−1^ at 0.2 C and maintained a reversible capacity of over 400 mAh·g^−1^ at a high rate of 4 C.

In future research, N/B co-doped graphene doping with the appropriate B atom possesses high research value, which plays a vital role in the capture of polysulfides. Moreover, N and B atoms doped graphene base materials with high conductivity and high specific surface area can maximize the cycle stability and cycle efficiency of LSBs. Therefore, how to frame the best percentage of the N/B atoms doping structure on graphene-based materials may be a research object that we are looking forward to in the future.

#### 3.2.3. N/O Dual Doped

Appropriate N/O co-doping has been proven to be beneficial to the anchoring of LiPSs. First, the S–O bond formed by doping makes the fixes the sulfur in the composite material firmly and inhibits the diffusion of the LiPS intermediates effectively. Second, N and O atoms carry an extra pair of electrons, which synergistically enhance the dipole moment and make it difficult to separate with Li atoms via dipole–dipole electrostatic interaction. N/O co-doping improves the surface polarity of the composite material so that the trapped polysulfide is bound to the cathode material, thereby improving the coulombic efficiency and cycle stability of the battery [90,91].

The N/O co-doped graphene nanotubes designed by Ogoke showed great potential in LSBs [92]. Graphene nanotubes have a large diameter. Physical encapsulation of LiPSs is the first step to prevent sulfur loss. Using transition metal nanoparticles to increase tube wall thickness and limit polysulfides to relieve free volume in the main structure space is the second step to prevent the diffusion of sulfur. The obtained sulfur cathode decorated with FeCoNi alloy (FeCoNi-GNT) showed a discharge capacity as high as 1234.7 mAh·g^−1^ under 0.05 C and a discharge capacity as high as 909.0 mAh·g^−1^ under 0.2 C. After 500 cycles of testing at a current rate of 1 C, the discharge capacity remained at 554.4 mAh·g^−1^. The nitrogen and oxygen content in S@FeCoNi-GNT are 4.7 and 3.0 at%. Although the content is not much, it can obviously promote electron transfer kinetics. Their results show that N and O co-doped graphene can be an effective way to improve the cycle stability of LiSBs. Recently, Shi et al. used N/O co-doped graphene by adding dents and micropores to the graphene sheets to fix the sulfur platelets [65]. This strategy can promote the rapid transport of Li^+^. The designed N, O co-doped graphene layered block (NOGB) and sulfur composite cathode (NOGB/S) preparation process are shown in Figure 7a. The discharge capacity of NOGB/S at 0.1 C was 1413 mAh·g^−1^, showed a high rate performance of 433 mAh·g^−1^ at 10 C, and was still maintained at 526 mAh·g^−1^ after 1000 cycles at 1 C.

So far, the N and O co-doped graphene system has been proven to be appropriate cathode material for LSBs and can exhibit good electrochemical performance. Utilizing the diversity of graphene substrates, enhancing the N and O co-doped graphene substrate materials through various physical packaging and chemical modifications are effective ways to achieve high-performance LSBs.

#### 3.2.4. Graphene Co-Doped with Single-Atom Metal and N

Transition metal particles have a significant catalytic effect in the field of graphene energy storage [93]. Increasing the polysulfide conversion rate through the doping of transition metal particles is also one of the important methods to improve the performance of LSBs. Since transition metals have special electronic orbital arrangements that non-metal elements do not have, the theoretical effect of using transition metals for graphene doping is even better [94]. Therefore, researchers have recently explored the use of transition metal and non-metal co-doped graphene as a suitable lithium-sulfur electrode.

Recently, mono-metal and N co-doped carbon have been used as nanocatalysts in oxygen reduction and hydrogen evolution [95,96,97,98,99,100]. Researchers have investigated the influence of monodisperse metal and N co-doping on the suppression of the shuttle, and used it in LSBs successively. As early as 2012, Luo et al. studied Li_2_S doped with transition metals in lithium battery cathodes, which opened the door to transition metal-doped graphene as lithium battery cathodes [101]. Zhang et al. used first-principles simulations to study the interaction between Fe and N co-doped graphene (FeN_4_@graphene) [89]. They found that a four-membered ring structure composed of sulfur atoms, iron atoms, lithium atoms and nitrogen atoms had a greater impact on the adsorption system. According to the Li_2_S_n_ (n = 1, 2, 4, 6, 8) system, the system can in turn be divided into several forms. From the specific structure of Figure 7d, it can be seen that Li_2_S has four four-membered rings and Li_2_S_2_ has two four-membered rings. According to research, the number of four-membered rings is directly proportional to the adsorption energy. Chen proposed that the Fe–S bond combination was due to charge transfer, which was different from the dipole–dipole combination of Li–N [95]. The charge transfer in the system mainly occurs near the four-membered ring. Fe and S have more extranuclear electrons, and the amount of charge transfer is larger than that of Li and N. Zeng et al. used density functional theory (DFT) to study the anchoring mechanism of FeN_x_ (x = 1, 2, 3, 4) and concluded that FeN_x_ is beneficial to the deposition and transformation of Li_2_S and Li_2_S_2_ during the discharge [102]. The above studies provide important theoretical support for Fe/N co-doped graphene. Based on the above theoretical research, Ji et al. prepared a Ni–N co-doped 3D graphene framework as cathode material for LSBs (S@Ni-N/G) [66], and its discharge capacity at 0.2 C was 1103.6 mAh·g^−1^, the capacity was stable at 953.5 mAh·g^−1^, and the coulombic efficiency was as high as 97% after 100 cycles.

The electrochemical performance of graphene doped with transition metals and non-metal elements is excellent when used in lithium-sulfur electrodes. The bond interaction of transition metals and graphene has a huge influence on LiPSs, but its theoretical research needs to be further improved. It is believed that the transition metals can be widely developed in the field of doped graphene electrodes through the continuous attempts and efforts of researchers.

## 4. Graphene-Based Composite Materials

Nowadays, more and more researchers tend to choose graphene composite materials as S electrodes. For example, the combination of metal compounds and graphene alleviates the shuttle effect from both physical and chemical aspects. The combination of other carbon materials and graphene allows them to perform their own duties, not only to obtain a composite network conductive structure support, but also to expand the pore area to improve the sulfur loading and sulfur utilization. The improvement of electrochemical performance is even more multifaceted.

### 4.1. Metal Compound/Graphene Composite

Inspired by the immobilization of LiPSs by single-atom chemical bonds, numerous studies have focused on the effect of polar metal compounds on the performance of the graphene electrodes. Metal particles such as cobalt, vanadium, and nickel are generally used as single-atom catalysts, and the high surface free energy of the metal center is used to catalyze polysulfides. However, due to the disadvantage that a single atom is easy to expose catalytic sites, it has to be introduced into materials with high specific surface area. The non-polar properties of graphene are a good choice for materials with large specific surface areas. The composite material of metal compounds and graphene is of great significance for suppressing the shuttle effect. Recently, Cui reported that bivalent metal oxides (MO) and tetravalent transition metal sulfides with M:O = 1:1 can maintain a high reversible capacity to promote battery electrochemical performance [103]. This research provides important insights into the design principles of transition metal compounds and graphene composite cathodes. The catalysis of polysulfide conversion is still in the early stages of research. This part mainly introduces the development of composite materials formed by different metal oxides, sulfides, and graphene.

#### 4.1.1. Metal Oxide

Oxygen vacancy (OV) defects are widely used in the field of catalysis because OVs can not only capture the adsorbent, but also inhibit the recombination of carriers. The O^2−^ state of the oxygen has a strong interaction with LiPSs, which reduces the amount of the migrated LiPSs [104,105,106,107,108]. Pt and Ni have been shown to have the ability to adsorb soluble polysulfides and can also accelerate the conversion of insoluble LiPSs to long-chain LiPSs and sulfur [109,110]. Consequently, adding metal oxides into graphene electrodes for catalysis is a feasible strategy.

**Figure 8:**
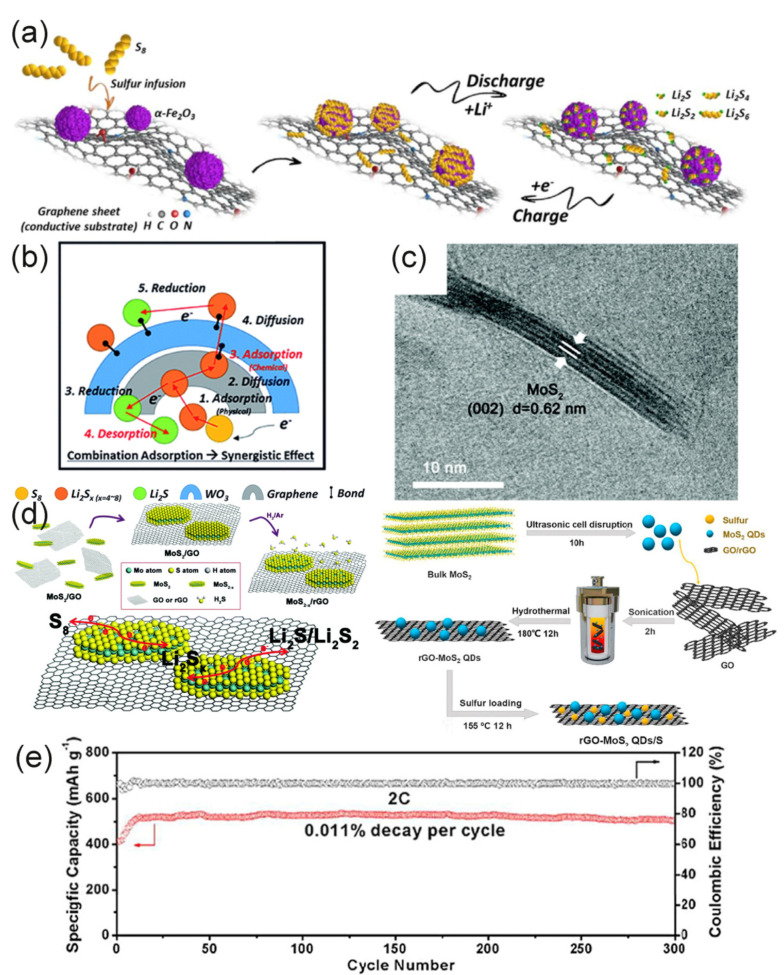
Metal compound/graphene composite sulfur cathode. (**a**) The Fe_2_O_3_ nanoparticles on the graphene sheet fix LiPSs by thermodynamic adsorption and promote the conversion of soluble LiPSs into insoluble LiPSs [112]. Copyright 2011, American Chemical Society. (**b**) WO_3_ in S@G@WO_3_ composites forms chemical bonds with LiPSs to inhibit the dissolution of LiPSs [113]. Copyright 2019, Royal Society of Chemistry. (**c**) HRTEM image of MoS_2_ nanosheets. (**d**) Schematic of the synthesis of the MoS_2__–__x_/rGO and rGO–MoS_2_ QDs/S. (**e**) Electrochemical performance of rGO–MoS_2_ QDs/S electrode [114,115]. Copyright 2019, Elsevier. Copyright 2017, Royal Society of Chemistry.

Tang used the advantages of metal oxides in the electrode preparation process [111]. Porous CaO is used as a CVD growth template to prepare graphene materials. CaO has two roles in this process: on one hand, CaO stimulates the rapid growth of graphene. On the other hand, it provides the electrode frame with medium- and large-pore sizes. This structure shortens the diffusion path of ions and also reduces interfacial resistance. In the electrochemical performance test, when the current rate was 0.5 C, the initial discharge capacity was 434 mAh·g^−1^, and the capacity decay rate for 150 cycles was 0.11% (coulomb efficiency was 90%). Zheng et al. prepared a three-dimensional Fe_2_O_3_–graphene hybrid (Fe–PGM) by a one-pot method [112]. It was found that α–Fe_2_O_3_ greatly enhances the interaction between the sulfur host and LiPSs by comparing the adsorption energy of S-containing clusters on graphene and α–Fe_2_O_3_. At the same time, it also promotes the conversion of soluble LiPSs to insoluble LiPSs during the charge and discharge process, which in turn improves the utilization rate of sulfur (Figure 8a). The maximum discharge capacity of the cathode compounded with sulfur (Fe–PGM–S) at 0.3 C is 1571 mAh·g^−1^ and the decay rate corresponding to 1000 cycles at a high current rate of 5 C is 0.049%. This also reflects the contribution of metal oxides in improving cycle stability.

Transition metal oxide films have received more attention in the field of sulfur cathodes due to their unique properties [116,117,118,119]. In consideration of the unique perovskite structure of tungsten trioxide (WO_3_) and excellent electrochromic, photochromic, and gasochromic characteristics, Choi combined WO_3_ film with graphene/sulfur nanomaterials (S@G@WO_3_) and used it in LSBs [113]. They found that WO_3_ could adsorb LiPSs with different chain lengths and concentrations during the redox reaction (Figure 8b). The use of WO_3_ film solves the problem of weak interaction between graphene and polysulfide, which makes the dissolution of polysulfide more difficult, thereby reducing the shuttle effect. Therefore, the cycle capacity is obviously increased (the capacity retention rate was 95% after 500 cycles).

Song et al. prepared vanadium dioxide/graphene sulfur cathode material (VO_2_/G/S), which possesses both trapping and catalytic effects [120]. VO_2_ has the advantages of high abundance, low cost, and rapid ion diffusion rate. In the VO_2_/G/S cathode, it showed strong anchoring of LiPSs, and the sulfur redox reaction was faster. It showed excellent electrochemical performance in the Li–S full battery. The initial discharge capacity at 0.2 C was 1405 mAh·g^−1^. Their research results provide a new way to prepare low-cost and environmentally-friendly Li–S cathodes. The proper polarity and high chemical stability of MoO_2_ are also make it a good choice of metal oxide as sulfur host materials. Feng et al. prepared one-dimensional hollow reduced graphene oxide-coated MoO_2_ nanotubes (H-S@MoO_2_/rGO) and achieved 84% high sulfur loading [121]. The special structure of nanotubes shortens the electron transmission path. The non-polar Mo–Mo metal bond in the MoO_2_ lattice exhibits metallic characteristics, and the combination with polarity increases the adsorption and charge transfer functions during the discharge process.

#### 4.1.2. Metal Sulfide

Metal sulfides have obvious advantages than metal oxides in LSBs. First, metal sulfides can reduce the lithiation voltage to avoid overlap with the operating voltage window; second, its conductivity is higher than that of metal oxides, which can significantly improve the utilization rate of the material. The third point is the redox reaction during the process, there is a strong interaction between metal sulfides and Li_2_S_x_, this can lower the barrier and catalyze the reaction process. The polar sites provided by metal sulfides inhibit the migration of polysulfides, at the same time, it can also be used as an active material for storing lithium to provide additional capacity. The addition of metal sulfides maximizes the catalysis and capture effects [27,28,33,81,122,123,124].

Since the introduction of the catalytic redox reaction strategy, researchers have tried to use various electrocatalysts to improve the electrochemical performance of materials. Yuan et al. mixed cobalt disulfide and graphene and used it in LiSBs [125]. As a conductive sulfiphilic host, CoS_2_ plays an indispensable role in the catalysis of redox reactions. Highly shrunken graphene was used as a substrate, and CoS_2_ clusters with a diameter of 1 μm were mechanically ground and mixed into it to obtain a CoS_2_/graphene composite (CoS_2_ + G). In the experiment of simulating a highly polar polysulfide as a statically adsorbed adsorbate, they found that the existence of aa Co–S bond greatly increased the affinity of Li_2_S_4_ molecules for heteropolar CoS_2_. When the CoS_2_ content was increased up to 30%, the adsorption effect of LiPSs in the electrolyte was not significantly enhanced. Therefore, CoS_2_ does not simply prevent polysulfides from penetrating into the electrolyte through chemical adsorption. Although this structure effectively improves the electron path, the small size of the CoS_2_ particles leads to insufficient distribution on the graphene framework. Thus, how to make up for the shortage of metal sulfides to further improve the performance of electrode materials should become one of the research hotspots in the future.

MoS_2_ is also a low-cost catalyst. Its high surface area and abundant active sites have attracted special attention from researchers. It has shown highly efficient catalytic ability in industrial reactions such as hydrogen evolution reaction (HER), oxygen reduction reaction (ORR), and oxygen evolution reaction (OER) [126,127,128,129]. Lin et al. decorated MoS_2_ nanoflakes on rGO [115]. As shown in Figure 8c, MoS_2_ nanoflakes have a structure of 6–8 layers, each layer is about 3–5 nm. MoS_2_ and RGO, as two-dimensional materials, have good contact compatibility, which not only improves the quality of the contact interface, but also successfully exposes the sulfur defects on the catalyst surface. This sulfur defect is an important factor in the catalytic conversion of polysulfides. When the sulfur cathode contained 4% MoS_2-x_/rGO, the capacity at an 8 C rate was 826.5 mAh·g^−1^. Moreover, 600 cycles could be carried out at 0.5 C rate and the capacity decay rate of each cycle was 0.083%. Both high rate performance and excellent cycle stability were obtained. The rGO-MoS_2_QDs (quantum dot)/S electrode prepared by Wei et al. through a hydrothermal reaction reached an ultra-low capacity decay rate of 0.011% per cycle in 300 cycles at the 2 C rate, and the coulombic efficiency was close to 100% (Figure 8e) [114]. Their preparation methods are similar, as shown in Figure 8d. Two kinds of MoS_2_ disulfide were obtained under the assistance of ultrasound and GO was prepared by the modified Hummer method [130], and finally, the sulfur cathode was prepared by the melt diffusion method. The difference between them was that when Lin synthesized MoS_2-x_/rGO, the amount of sulfur deficiency was changed by changing the heat treatment time and temperature in hydrogen. However, Wei et al. directly made the mixture solution undergo a hydrothermal reaction and then freeze-drying, which did not change the situation of sulfur deficiency [114]. These studies confirm the effectiveness of MoS_2_ as a polysulfide conversion catalyst.

In recent years, nickel sulfide (Ni_3_S_2_) has also received attention in supercapacitors. Ni_3_S_2_ is not only low-cost but is also highly conductive. Guo et al. synthesized a Ni_3_S_2_/(N, S)-rGO hybrid material [131]. The excellent effect brought by the N/S co-doping modification above-mentioned was still effective. The co-doping of Ni_3_S_2_ and N/S synergistically improved the material’s conductivity and polysulfide adsorption capacity. Both chemical bonding and catalytic effects were reflected. The recombination with a 3D-rGO structure suppressed the shuttle effect from both physical and chemical aspects. The composite with 28.2 wt% Ni_3_S_2_ content showed good performance in the charge–discharge test. A total of 1000 cycles were reached at a current density of 3 C, and the capacity decay rate per cycle was 0.023%. When the sulfur loading density per unit area was as high as 5.8 mg·cm^−2^, the capacity remained above 72.5% after 200 cycles at 1 C.

### 4.2. Other Carbon Materials/Graphene Composite Materials

The graphene-based material as the sulfur host does provide a large specific surface area for the electrode and sufficient space for sulfur loading. However, the long-term electrostatic attraction inside the graphene sheets makes the graphene sheets continuously gather and accumulate, which destroys the large specific surface area of sulfur loading to a certain extent. The specific capacity and rate capability also gradually decrease [132,133]. Along with the self-supporting carbide-derived carbon/CNT/S composite cathode first proposed by the Kaskel group, the advantages of the conductivity and cycle stability of this dual carbon material mixture have gradually been recognized by researchers [134]. As such, researchers’ attention to the dual carbon material mixture has risen to a new level.

Carbon nanotubes (CNTs) are multi-purpose matrix materials with high conductivity and large specific surface area. However, CNTs have limited performance as a conductive matrix. Both CNT and graphene are representative carbon allotropes, and their basic structural units are hexagonal honeycomb lattices of carbon. CNT is a one-dimensional nanotube structure. It is not limited by the area of the active material and CNTs with a large aspect ratio can promote the rapid transmission of lithium ions and electrons. Graphene is a two-dimensional nanosheet. Its planar structure will prevent the transmission of lithium ions, which is particularly obvious at high current densities [31,135,136]. From the analysis of material surface properties, graphene had a rich pore structure and more surface functional groups and defects. The theoretical specific surface area was 2630 m^2^·g^−1^. The various atomic doping behaviors described in the previous sections are important factors that limit the diffusion of polysulfides. The rich pore structure also helps electrolyte penetration. CNTs are usually obtained by CVD, and the lack of surface functional groups can be attributed to the high purity [137]. Of course, a large number of functional group modifications will inevitably lead to a decrease in the conductivity of graphene. Thus, the advantages of CNTs will emerge at this time. The above analysis indicated that graphene and CNTs have complementary characteristics in many aspects. Rather than treating them as competitors, it is better to combine them cleverly to exert beneficial synergies. Breaking their limited characteristics by constructing a CNT/graphene hybrid structure is an effective method.

Graphene/single-walled CNT (SWCNT) hybrid materials are considered to have superior performance in constructing conductive networks than other sp^2^ nano carbons [39,138]. Such G/SWCNT preparation strategies usually choose layered double hydroxides (LDHs) [139] or layered double oxides (LDO) [140] as the 2D lamellar substrates for graphene CVD growth to achieve the inherent position of SWCNT and graphene. This method usually chooses methane as the growth carbon source and removes the calcined LDH or LDO after the high-temperature CVD process to obtain a G/SWCNT hybrid. The hybrid prepared by this method anchors SWCNTs on the surface of the graphene layer, thereby inhibiting the aggregation of SWCNTs. The G/SWCNT–S cathode prepared by Zhao had a capacity of 650 mAh·g^−1^ after 100 cycles at a current rate of 5 C, which proves the feasibility of this scheme [139].

**Figure 9:**
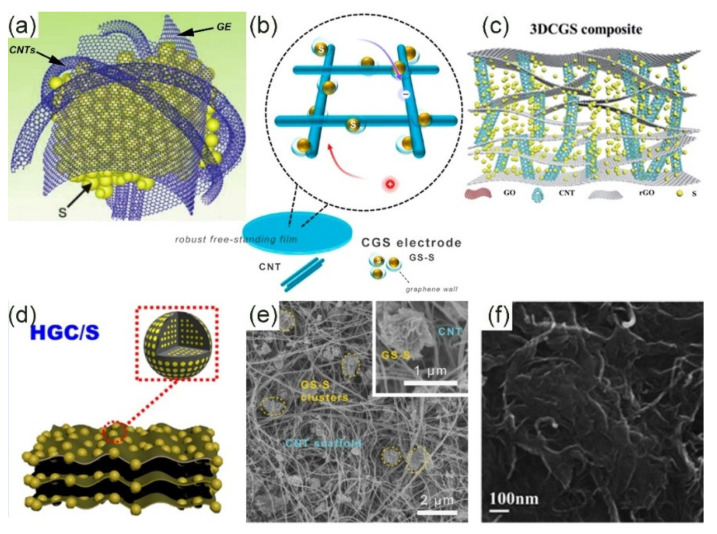
Various carbon/G composite cathode configurations. (**a**) Schematic diagram of G/CNT hybrid [142]. Copyright 2017, John Wiley and Sons. Schematic diagram of ultra-long CNT/GS-S structure (**b**) and its SEM image (**e**) [141]. Copyright 2015, Elsevier. 3DCGS composite cathode structure (**c**) and its SEM image (**f**) [143]. Copyright 2015, Royal Society of Chemistry. (**d**) Graphene and carbon nanospheres structure diagram [144]. Copyright 2019, Elsevier.

In the initial studies, researchers started to combine graphene and CNTs from the basic shape. For example, the combination of ultra-long CNT and graphene spheres (GS) (Figure 9b) [141], graphene and CNT co-growth seamlessly symbiosis model (Figure 9a) [142], and the combination of the three-dimensional sponge-like morphology of ordinary CNT and graphene [143]. Ultra-long CNTs have excellent flexibility and can also provide a highly conductive network, providing an effective way for flexible electronic devices with high energy density. The internal size of hollow graphene nanospheres is about 15–30 nm, which has dual functions: on one hand, it provides a tight load space for active sulfur; on the other hand, it maintains volume fluctuations during the cycle to prevent the dissolution of LiPSs (Figure 9e) [141]. A three-dimensional CNTs/graphene-sulfur (3DCGS) sponge structure prepared by He et al. enabled the sulfur loading to reach 80.1% [143]. CNTs were used to enhance conductivity and adjust the mesoporous structure. Compared with the three-dimensional graphene-sulfur (3DGS) sponge without carbon nanotubes, the conductivity was increased by 324.7%. Furthermore, of prominence is that the pore size in 3DG was only 3.5 nm, and 3DCG formed a new mesopore with a size of 32.1 nm, so a large amount of active sulfur was incorporated into it. The electrolyte permeability also improved greatly (Figure 9c,f). The capacity of the entire electrode at a high rate of 4 C was 653.4 mA·hg^−1^.

The amount and form of loading sulfur are also some of the evaluation criteria for electrode performance. For GO/CNT/S prepared by the freeze-drying method, Hwa first added sulfur and its sulfides during the electrode preparation process and finally removed the impurities [19], while Gómez-Urbano first completed the preparation of GO-CNT and finally added S through the melt diffusion method [145]. In the former, due to the advantages of the preparation process and the clever embedding of CNTs, there was no obvious S agglomeration when the S content was as high as 87%. The latter electrode with a sulfur loading exceeding 4.0 mg·cm^−2^ had a better specific capacity when doped with 2 wt% CNT (the specific capacity value was 500 mAh·g^−1^ after 100 charge/discharge cycles at 0.1 C).

The abundant functional groups on graphene are also one of the breakthrough points of G/CNT composites. Combined with the heteroatom-modified graphene structure mentioned in previous sections, single-atom doping or diatomic co-doping are both effective ways to improve the adsorption performance of G/CNT. For example, Su et al. converted Prussian blue (dehydrated sodium ferrocyanide) into a N-doped graphene-carbon nanotube hybrid material through one-step pyrolysis [142]. The active sites generated by nitrogen doping effectively trap polysulfides, greatly improving the cycle performance. Wen et al. prepared N-GCNT composites using a one-step ultrasonic spraying deposition method and successfully penetrated N atoms into the carbon lattice [146]. The interaction between N and S atoms limited the dissolution of LiPSs. Interestingly, the dopant atoms here are effective for the modification of carbon materials. Wu et al. used egg white as a precursor for the inherent N/P co-doping [147]. This preparation method was economical and environmentally friendly. N/P elements were uniformly doped on the carbon framework and the chemical adsorption capacity of the composite material was improved. The N-doped carbon/graphene sheet was designed by Xu et al. and used as cathode material for fixing sulfur [148]. According to reports, the shuttle of polysulfides was still greatly inhibited in the cathode without LiNO_3_ in the electrolyte. Lee et al. prepared a graphene-loaded N-doped carbon framework (NCF-G) [149]. The initial capacity of the NCF-G electrode compounded with sulfur at 0.1 C was 1359.7 mAh·g^−1^. Sun et al. decorated graphene with N and S co-doped carbon nanowalls (NSCNWs) to effectively trap polysulfides [150]. The NSCNW-G/S cathode exhibited an ultra-low capacity decay rate (the capacity decay rate for 100 cycles at 0.2 C was 0.078%). The above studies of heteroatom-modified dual carbon materials have shown their practicality for battery performance.

In addition to the CNT structure, electrodes with spherical structures have also been studied accordingly. The nanosphere structure can hold a large amount of sulfur and increase the contact area with the electrolyte to promote the transport of Li^+^. The most important thing is that it can fix polysulfides well. Zhou et al. designed N-doped double-shelled hollow carbon spheres (G–NDHCS–S) coated with graphene to capture sulfur [132]. Due to the highly electronic conductive network provided by the graphene package, the material did not need to add additional conductive additives and binders. The design of the porous double-shelled hollow structure significantly improves the electrochemical performance of lithium-sulfur batteries. The dual-confined flexible cathode configuration wrapped with carbon spheres and graphene enables the initial discharge capacity to reach 1360 mAh·g^−1^ at a current rate of 0.2 C. The graphene/carbon nanosphere composite material synthesized by Jia et al. possessed a specific surface area of 3200 m^2^·g^−1^ [144]. Its structure is shown in Figure 9d. This structure made the sulfur content 74.5 wt%. The layered porous structure and macropore volume could still effectively fix sulfur. The electrode still had a capacity of 916 mAh·g^−1^ after 100 cycles. Therefore, the potential performance of the spherical structure electrode in the LSBs has a very broad development prospect.

Additionally, current collectors based on other carbon materials and graphene have shown multiple advantages in LSBs. First, they show excellent flexibility. This is particularly prominent in CNT/graphene electrodes and provides an important foundation for the research of flexible batteries. Second, they mostly act as porous containers to hold polysulfides during the oxidation–reduction reaction process and improve the cycle stability of the battery.

## 5. Conclusions and Outlook

In summary, this article took graphene as a theme and summarized the potential applications of the pure graphene framework, heteroatom-modified graphene framework, and graphene composite framework in LSB cathodes in detail, aiming at obtaining LSBs with high energy density and long cycle capability and promoting their long-term development and application. The idea of graphene as a sulfur cathode carrier originates from its high conductivity, large specific surface area, and strong adsorption capacity for LiPSs on a surface defect. This viewpoint has also been confirmed by the composite structure of sulfur and the simple graphene framework. Sulfur cathodes have been designed with various structures such as sandwich, core-shell, and hybrid structures. Due to the flexibility and controllable assembly capabilities of graphene, the electrochemical performance of LSBs has improved rapidly. In the field of heteroatom-modified graphene cathodes, researchers gradually discovered that pyridinic-N had stronger chemisorption energy than other forms. It is worth noting that in addition to chemical bonding, transition metal doping also exhibits additional electrocatalytic capabilities. In order to solve the problem of limited content of single atom doping, researchers have gradually used diatomic doped graphene to achieve the common capture of LiPSs by a variety of chemical bonds. Metal compound additives have two main functions in the S electrode: one is to fix polysulfides, and the other is to catalyze redox reactions to promote polysulfide conversion. A small number of metal compounds can also serve as active materials to store lithium, increasing the lithium storage capacity. The advantage of the composite of other carbon materials and graphene is to achieve a diversified conductive network structure, thereby improving the conductivity of the skeleton, increasing the sulfur content and expanding the pore volume.

However, although many efforts have been made to achieve better electrochemical performance, there are still many new challenges in LiSBs. For instance, although the stronger bonding force of pyridinic-N has been confirmed through repeated experiments, accurate control of the type and content of nitrogen doping cannot be achieved. The cost of boron doping is high, the doping process is complex, and the accuracy of characterization is difficult. The theoretical system for the interaction of transition metals and graphene bonds is not yet clear. There are also many challenges in the field of heteroatom doping. For metal additives, although catalytic conversion and effective chemical bonding inhibit the diffusion of polysulfides, the conductivity of such additives is not ideal, which inevitably reduces the battery capacity and cycle capacity. In this respect, the conversion of the catalyst into quantum dots may produce unexpected performance results.

For graphene substrate materials, the key to improve the electrochemical performance of LSBs is the dissolution of polysulfides. The inhibiting of polysulfide diffusion has mainly focused on the physical properties of macroscopic physical encapsulation and microscopic van der Waals forces. In terms of chemical properties, it has the function of chemical bonding and catalysis, which accelerates the reaction process. The combination of physical action and chemical action is expected to be the most effective way of inhibition. This is a long way toward the industrial production of lithium-sulfur batteries, and it is necessary to coordinate development in many aspects such as the preparation process, applicable environment, high energy density, and economic benefits to promote the commercial application of LSBs.

## Figures and Tables

**Table 1:** Electrochemical performance of atom-modified graphene-based sulfur cathode.

Dopant Atom	Sulfur Host	Sulfur Content	Main Bonds	Doping Content (at.%)	Initial Capacity/Rate (mAh·g^−1^/C)	Retain Capacity/Cycles	Ref.
N	S@N-3D-rGO	80 wt%	C–C/C=C/C–O/C=O/C–N	-	1042/0.2	94.8%/100	[54]
	3D RNGO/S	90 wt%		-	1186/0.1	96%/200	[55]
	NG/S	74 wt%		6.89	1309/1	663 mAh·g^−1^/300	[56]
	N-GA1/S	75.5 wt%		9.23	1210.7/0.1	72.4%/50	[57]
B	BGA-S	59 wt%	−BC_2_O, −BCO_2_, −BC_3_	1.76	1290/0.2	994 mAh·g^−1^/100	[58]
	S@BEEG	72.5 wt%		1.86	1476/0.1	82%/130/1 C	[59]
N, S	NSG-4/S	68 wt%	C–N/C–S/S–O/S–S	N: 2.47 S: 6.31	1583/0.1	819 mAh·g^−1^/100	[60]
	3D N, S-GP/S	8.5 mg·cm^−2^		-	1200/0.2	63%/500/0.5 C	[61]
	NSG/S	43.3 wt%		N: 5.99 S: 5.89	1433/2	684 mAh·g^−1^/200	[62]
	A-NSG@S	72.4 wt%	–C=S–/C–N/C–S/C=N	N: 4.18 S: 0.85	1178/0.2	780 mAh·g^−1^/600	[63]
N, B	NBCGN/S	65 wt%	−BC_2_O/−BCO_2_/ −BC_3_/B_2_O_3_/B-N	N: 6.6 B: 7.0	1200/0.2	76%/300	[64]
N, O	NOGB/S	76 wt%	–COOC–/C–OH/C=O/	N: 3.0 O: 18.1	1413/0.1	526 mAh·g^−1^/1000/1 C	[65]
Ni, N	S@Ni-N/G	2.0 mg·cm^–2^	Ni–N/Ni–C/Ni–Ni/Ni–S	-	1103.6/0.2	953.5 mAh·g^−1^/100	[66]

## Data Availability

No new data were created or analyzed in this study. Data sharing is not applicable to this article.
